# Prenatal versus postnatal sex steroid hormone effects on autistic traits in children at 18 to 24 months of age

**DOI:** 10.1186/2040-2392-3-17

**Published:** 2012-12-11

**Authors:** Bonnie Auyeung, Jag Ahluwalia, Lynn Thomson, Kevin Taylor, Gerald Hackett, Kieran J O’Donnell, Simon Baron-Cohen

**Affiliations:** 1 Autism Research Centre, Department of Psychiatry, University of Cambridge, Douglas House, 18B Trumpington Rd, Cambridge CB2 8AH, UK; 2 Neonatal Service, Rosie Maternity Hospital, Robinson Way, Cambridge, CB2 0QQ, UK; 3 Department of Clinical Biochemistry, Addenbrooke’s Hospital, Cambridge, CB2 0QQ, UK; 4 Department of Fetal Medicine, Rosie Maternity Hospital, Robinson Way, Cambridge, CB2 0QQ, UK; 5 Douglas Mental Health University Institute, McGill University, Montreal, QC, Canada

## Abstract

**Background:**

Studies of prenatal exposure to sex steroid hormones predict autistic traits in children at 18 to 24 and at 96 months of age. However, it is not known whether postnatal exposure to these hormones has a similar effect. This study compares prenatal and postnatal sex steroid hormone levels in relation to autistic traits in 18 to 24-month-old children.

Fetal testosterone (fT) and fetal estradiol (fE) levels were measured in amniotic fluid from pregnant women (n = 35) following routine second-trimester amniocentesis. Saliva samples were collected from these children when they reached three to four months of age and were analyzed for postnatal testosterone (pT) levels. Mothers were asked to complete the Quantitative Checklist for Autism in Toddlers (Q-CHAT), a measure of autistic traits in children 18 to 24 months old.

**Finding:**

fT (but not pT) levels were positively associated with scores on the Q-CHAT. fE and pT levels showed no sex differences and no relationships with fT levels. fT levels were the only variable that predicted Q-CHAT scores.

**Conclusions:**

These preliminary findings are consistent with the hypothesis that prenatal (but not postnatal) androgen exposure, coinciding with the critical period for sexual differentiation of the brain, is associated with the development of autistic traits in 18 to 24 month old toddlers. However, it is recognized that further work with a larger sample population is needed before the effects of postnatal androgen exposure on autistic traits can be ruled out. These results are also in line with the fetal androgen theory of autism, which suggests that prenatal, organizational effects of androgen hormones influence the development of autistic traits in later life.

## Background

Autism Spectrum Conditions (ASC) occur more often in males than females [1]. This sex difference may provide an important clue to the etiology of the condition, though the true sex ratio in ASC is unclear given the known difficulties in the diagnosis of ASC in females [2]. ASC have strong neurobiological and genetic components [3]; however, the specific factors (hormonal, genetic or environmental) that are responsible for the higher risk of ASC in males remain unclear.

Hormones perform critical functions in both neural and physical development throughout life, from pregnancy to adolescence and beyond [4]. There are several lines of evidence that suggest that prenatal exposure to hormones, in particular fetal testosterone (fT), play a role in the development of ASC. This includes the finding that elevated fT is related to reduced eye contact [5], slower language development [6], poorer quality of social relationships and narrower interests [7], and reduced empathy [8]. Higher fT levels also predict increased systemizing ability [9], and more autistic traits in school-age children [10] and in toddlers [11].

To date, no studies have tested if there is also a link between postnatal testosterone and the development of autistic traits in later life. A possible window of interest lies between three and four months of age, when a second peak in testosterone levels is thought to occur [12]. This second peak may play a significant role in physical [13] and behavioral development [14]. In addition to testosterone, the estrogen hormone estradiol is another hormone that is synthesized prenatally from testosterone. Estradiol is considered to be the most biologically active estrogen [15].

The aim of this study is to compare if prenatal and postnatal sex steroid hormone levels in the same individuals are associated with differences in autistic traits at 18 to 24 months of age, using the Quantitative Checklist for Autism in Toddlers (Q-CHAT).

## Methods

### Participants

Mothers were asked for consent to participate in research at the time of having an amniocentesis. Medical records of approximately 700 patients who had undergone amniocentesis in the Cambridge region between January 2004 and July 2006 were examined. Participants were excluded if: (a) amniocentesis revealed a chromosomal abnormality; (b) the pregnancy ended in miscarriage or termination; (c) the child suffered neonatal or infant death; (d) the child suffered significant medical problems after birth; (e) there was a twin pregnancy or (f) the relevant information was absent from medical records. General Practitioners were asked for consent to contact the women, and mothers with babies less than four months of age were invited for participation (n = 104). This study was given ethical approval by the National Health Service Suffolk Research Ethics Committee. Written informed consent was obtained from General Practitioners and participating parents.

A total of n = 47 (22 boys, 25 girls) mothers brought their children in for saliva collection, where a sample of passive drool was taken when their child reached three-months of age. Twelve samples had insufficient amounts of saliva, with a total of 35 samples (15 boys, 20 girls) remaining that were eligible for analysis. Saliva was collected using a suction machine under the supervision of a trained health professional. The families were contacted when their child reached 18 months, and were asked to complete the Q-CHAT [16]. Parents were blind to both the fetal and postnatal hormone levels of their child.

### Outcome variable

The Quantitative Checklist for Autism in Toddlers (Q-CHAT) is a 25-item parent-report screening measure developed to identify toddlers at risk for the development of ASC [16]. A total score is obtained with a minimum score of 0 and a maximum score of 100 [16].

### Hormone measurements

Fetal testosterone (fT) and fetal estradiol (fE) levels were measured by radioimmunoassay in amniotic fluid. See Auyeung *et al.* (2010) for details. Postnatal testosterone (pT) levels were assayed (without separation or extraction) for testosterone from saliva samples using commercially available immunoassay protocols (Salimetrics, State College, PA, USA). Average intra- and inter-assay coefficients of variation were 10% and 15%, respectively. A serial dilution was performed (2.5) to give a standard curve with greater sensitivity at lower ranges.

### Demographic variables

Gestational age at amniocentesis (in weeks), maternal age, parental education level, presence of older siblings and the child’s age were included as control variables.

## Finding

Levels of fE were positively skewed (skewness >1) so natural logarithmic transformed data were used in subsequent analyses. Distributions of the other variables (including fT) were not skewed. The ranges of the Q-CHAT scores obtained in this sample were consistent with scores from a large sample of typically developing children [16]. See Table 1 for descriptive information and Table 2 for correlations.

**Table 1:** Descriptive statistics

	**Combined group**				**Girls**				**Boys**				
**Variable**	**n**	**M**	**SD**	**Range**	**n**	**M**	**SD**	**Range**	**n**	**M**	**SD**	**range**	**Cohen**’**s D**
fT level (nmol/L)**	35	0.58	0.45	0.05 to 2.28	20	0.34	0.23	0.05 to 0.95	15	0.91	0.47	0.42 to 2.28	1.54
fE level (nmol/L)	35	0.28	0.18	0.14 to 1.15	20	0.24	0.08	0.14 to 0.45	15	0.34	0.25	0.18 to 1.15	0.54
pT level (nmol/L)	35	0.17	0.06	0.02 to 0.29	20	0.18	0.07	0.02 to 0.29	15	0.16	0.05	0.04 to 0.23	0.31
Gestational age (weeks)	35	16.70	1.47	13 to 21.2	19	16.62	0.90	15 to 18.1	14	16.82	2.04	13 to 21.2	0.13
Child age (months)	35	19.34	2.93	18 to 35	20	19.10	1.33	18 to 23	15	19.67	4.27	18 to 35	0.18
Maternal age (years)	35	36.54	4.74	21 to 46	20	37.75	3.84	33 to 46	15	34.93	5.44	21 to 45	0.60
Parental education (1–5)	35	3.41	0.90	1.5 to 5	20	3.30	0.89	1.5 to 4.5	15	3.57	0.92	2 to 5	0.30
Q-CHAT score**	35	27.03	8.62	10 to 43	20	23.95	8.62	10 to 43	15	31.13	6.94	14 to 40	0.92

*Indicates raw values.

* Sex difference is significant at the *P* <0.05 level.

** Sex difference is significant at the *P* <0.01 level.

**Table 2:** Correlation matrix for all cases

	**fT level**	**fE level**	**pT level**	**Sex**	**Gest**. **age**	**Child age**	**Matr**. **age**	**Parent ed**	**Older sister**	**Older brother**
fE Level	.03	--	--	--	--	--	--	--	--	--
pT Level	.13	-.11	--	--	--	--	--	--	--	--
Sex	.72**	.28	-.13	--	--	--	--	--	--	--
Gestational age	.17	-.47**	.10	.04	--	--	--	--	--	--
Child age	-.08	.22	-.21	-.09	-.10	--	--	--	--	--
Maternal age	-.40**	-.09	-.18	-.28	.10	.10	--	--	--	--
Parent education	.03	.14	.31	.09	-.12	.02	.01	--	--	--
Older sister	-.12	-.18	-.09	-.14	.02	.38	.32	-.07	--	--
Older brother	-.08	.05	-.31	.02	-.10	.18	-.04	.17	.03	--
Q-CHAT score	.56**	.17	-.09	.46**	-.10	.18	-.32	.09	-.01	.15

* *P* <0.05.

** *P* <0.01.

Due to the small sample sizes, nonparametric statistics were used to examine sex differences and correlation values. Mann–Whitney U tests showed no significant sex differences in pT levels (Mann–Whitney U = 127.5, *P* >0.05) or fE levels (Mann–Whitney U = 101.0, *P* >0.05). Significant sex differences were found for fT levels (Mann–Whitney U = 24.0, *P* <0.001) and for Q-CHAT score (Mann–Whitney U = 70.5, *P* <0.01). fT level and sex were also significantly related (Spearman’s rho = 0.72, *P* <0.001). However, no relationships between fT levels and fE levels (Spearman’s rho = 0.03, *P* >0.05) were found. In addition, no relationship was observed between pT levels and sex (Spearman’s rho = −0.13, *P* >0.05), between fT and pT levels (Spearman’s rho = 0.13, *P* >0.05) or between pT levels and fE levels (Spearman’s rho = −0.11, *P* >0.05). A significant relationship was observed between fE levels and gestational age, but neither of these variables were related to Q-CHAT scores.

**Figure 1:**
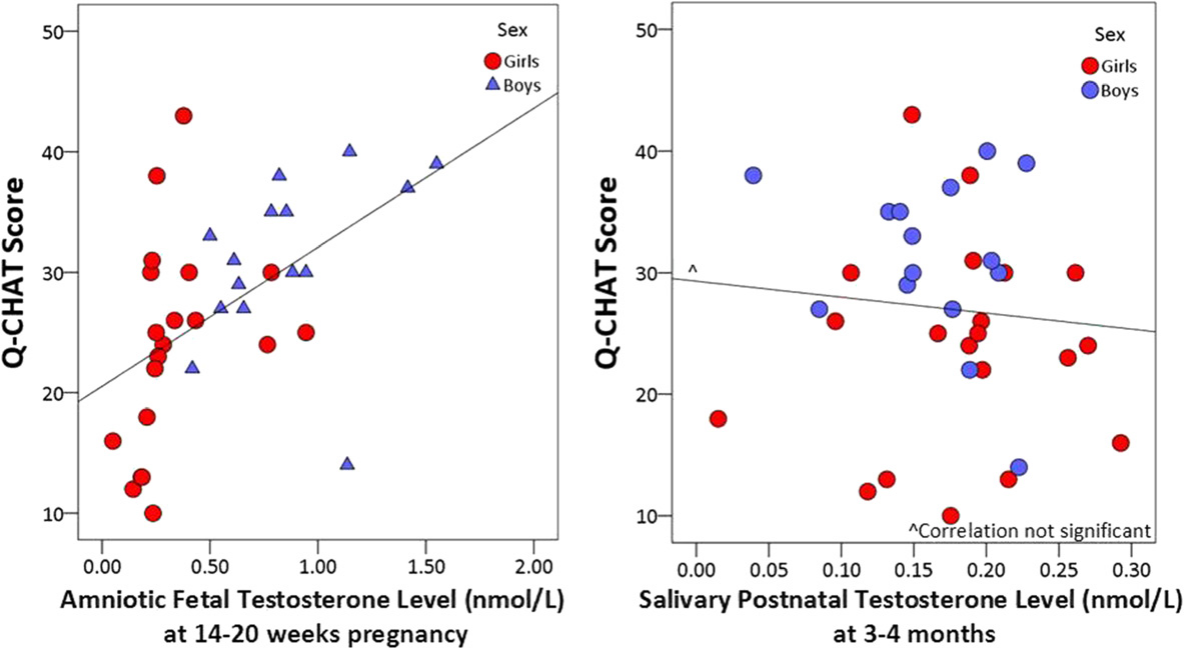
**fT and pT levels and Q**-**CHAT scores.**

A hierarchical multiple regression analysis was used. In the first stage any predictor variable showing a significant correlation with the outcome at *P* <0.20 was included using the enter method [17]. Maternal age was the only variable that met the *P* <0.20 criterion for entry into the first stage (Spearman’s rho = −0.32, *P* <0.20). The presence of suppressor variables (predictors that are highly correlated with other predictors at *P* <0.01) was investigated. However, no suppressor variables were observed. The main effects of fT level, pT level and child sex were tested for inclusion in the second stage using the stepwise method (entry criterion *P* <0.05, removal criterion *P* >0.10). The interaction between child sex, fT level and the fT/pT interaction were tested for inclusion in the third stage using the stepwise method (entry criterion *P* <0.05, removal criterion *P* >0.10). fT level was the only variable included in the final regression model and produced a significant F-change (F-change = 8.82, *P* <0.01, ΔR^2^ = 0.21). Table 3 shows the regression results for boys and girls together. See Figure 1 for plots of the relationships between fT and Q-CHAT scores and pT and Q-CHAT scores.

**Table 3:** **Final regression model for Q**-**CHAT scores**

		**Final regression model**					
**Outcome**	**Predictors**	**R**^**2**^	Δ **R**^**2**^	**B**	**SE**	**β**	**Sig**
Group							
Q-CHAT Score	Maternal age	0.05	0.05	0.07	0.30	0.04	*P* >0.05
	fT level	0.26	0.21	11.20	3.77	0.49	*P* <0.001

Within sex correlations are shown in Table 4. A significant correlation between fT levels and Q-CHAT scores was found for both boys (Spearman’s rho = 0.51, *P* <0.05) and girls (Spearman’s rho = 0.57, *P* <0.05). No significant relationships between fE, pT or any other demographic variables and Q-CHAT scores were found in boys or girls.

**Table 4:** Correlation matrix for girls (above diagonal) and boys (below diagonal)

	**fTlevel**	**fElevel**	**pT level**	**Gest**. **age**	**Child age**	**Matr**. **age**	**Parent ed**	**Older sister**	**Older brother**	**Q**-**CHAT score**
fT Level (nmol/L)	--	−0.32	0.22	0.45	0.20	−0.14	0.10	0.25	0.09	0.57*
fE Level (nmol/L)	0.10	--	−0.40	−0.50*	0.41	0.13	0.17	−0.13	0.31	0.09
pT Level (nmol/L)	0.43	0.44	--	0.46*	−0.30	−0.41	0.60*	−0.31	−0.21	−0.01
Gestational Age	−0.19	−0.54*	−0.27	--	−0.29	0.00	0.17	0.02	−0.27	−0.21
Child Age	−0.22	−0.04	−0.10	0.21	--	0.13	0.13	0.39	0.24	0.35
Maternal Age	−0.44	−0.20	−0.04	0.30	−0.03	--	−0.13	0.10	−0.23	−0.03
Parent Education	−0.13	−0.07	−0.15	−0.37	−0.13	0.29	--	−0.16	0.18	0.18
Older Sister	−0.48	−0.16	0.27	0.10	0.26	0.59	0.19	--	−0.07	0.12
Older Brother	−0.38	−0.38	−0.42	0.08	0.12	0.25	0.13	0.21	--	0.33
Q-CHAT Score	0.51*	−0.23	−0.09	0.04	−0.13	−0.39	−0.19	−0.27	−0.05	--

* *P* <0.05.

## Discussion

This study examined relationships among fetal testosterone (fT), estradiol (fE), postnatal testosterone (pT) and scores on the Quantitative Checklist for Autism in Toddlers (Q-CHAT). Results confirmed earlier findings that sex differences in autistic traits are present in toddlers, with boys scoring much higher than girls [11,16]. fT level was the only variable which was significantly related to Q-CHAT scores when both sexes were combined. However, no association was found between fT levels and fE levels, between Sex and fE levels (r = −0.02, *P* >0.05) or between Sex and pT levels. A correlation between fE levels and gestational age was found. However, neither of these variables was related to Q-CHAT scores suggesting that it is fT rather than fE or gestational age that predicts Q-CHAT scores. Levels of postnatal testosterone at three to four months of age were also 70% lower than in the second trimester and did not show a significant effect on Q-CHAT score at 18 to 24 months.

In a regression model, fT level was the only predictor retained in the final stage, whereas both Sex and the fT/Sex interaction were excluded; suggesting this could be an effect of fT, rather than Sex. Within Sex findings also show significant correlations between Q-CHAT score and fT level in boys and in girls. This corresponds with related findings in larger samples both within this age group and in older children [10,11].

The relationship observed between Q-CHAT scores and fT level (but not pT level) provides preliminary support for the notion that fetal rather than postnatal exposure to testosterone is critical for the development of autistic traits in toddlers. However, it is recognized that this study has a limited sample size and that postnatal testosterone should not be discounted as a factor in the development of autistic traits until further testing of these associations in a larger sample of children.

## Conclusions

The current study examined the link between prenatal and postnatal hormone levels and later autistic traits. The Q-CHAT is a validated measure of autistic traits, which has been shown to differentiate toddlers who go on to receive an ASC diagnosis later in childhood. Results showed that fT levels are positively related to Q-CHAT scores in this sample of typically developing children. The lack of any relationship between pT or fE levels and other variables (including Sex) indicates that fetal exposure to testosterone has a greater influence on the development of autistic traits than exposure to the same hormone after birth. The relationship between fT levels and Q-CHAT scores remains consistent with previous findings, despite the smaller sample of children in this study. However, findings relating to pT require validation in a larger study. Although higher levels of fT are unlikely to be the sole cause of autism, this work aids our understanding of the potential mechanisms that could contribute to the development of such conditions. These results are in line with the fetal androgen theory of ASC [18], and a large-scale collaborative project to test the role of prenatal androgens in ASC is underway.

## Abbreviations

ASC: Autism Spectrum Conditions; fE: Fetal estradiol; fT: Fetal testosterone; pT: Postnatal testosterone; Q-CHAT: Quantitative Checklist for Autism in Toddlers.

## Competing interests

The authors declare that they have no competing interests.

## Authors’ contributions

BA conceived of and carried out the study and drafted the manuscript. JA and LT enabled the follow-ups of the infants. KT carried out the amniotic fluid radioimmunoassays. GH participated in the recruitment of women who had had an amniocentesis. KJO carried out the salivary immunoassays. SBC contributed to the study design and drafting of the manuscript. All authors read and approved the final manuscript.
